# Prevalence of cell phone use while driving in different urban settings. A roadside observational study in maputo city, Mozambique

**DOI:** 10.1016/j.heliyon.2025.e42047

**Published:** 2025-01-16

**Authors:** Alfredo Júlio Maposse, Lucie Laflamme, Franziska Fischer, Jette Möller

**Affiliations:** a Eduardo Mondlane University, Department of Psychology, Centre of Studies and Psychological Support (CEAP), Maputo, Mozambique; b Karolinska Institutet, Department of Global Public Health, Stockholm, Sweden; c University of South Africa, Institute for Social and Health Sciences, Pretoria, South Africa

**Keywords:** Distracted driving, Smartphone, Crossings, Sub-saharan africa, Car drivers, Three-wheelers

## Abstract

**Introduction:**

All forms of cell phone use while driving poses a threat to traffic safety. Few studies have reported the prevalence from low- and middle-income countries where the risk of severe road traffic crashes is highest. This study aimed to ascertain the prevalence of cell phone use among motor vehicle drivers in Maputo city, Mozambique, considering different types of vehicles and cell phone use.

**Methods:**

A roadside observational study was conducted to assess cell phone use while driving in three typical urban environments (on straight roads, at intersections, and by roadside markets) during three daytime periods. The prevalence of use, expressed in percentages with 95 % confidence intervals, was estimated by type of vehicle and sex.

**Results:**

An overall 9.3 % prevalence of cell phone use was observed among the 11 680 completed observations of motor vehicle drivers. Prevalence was lower at intersections and by roadside markets (7.1 % respective 7.5 %) and higher on straight roads (12.9 %) and, for men, three times as high among car drivers (10.3 %) than among drivers of motorized three-wheelers (3.3 %). Speaking/listening was more prevalent than texting/reading for both women (52.6 % vs 36.1 %) and men (46.4 % vs 40.5 %). At all three types of sites, men in trucks spoke/listened more than men in other type of vehicles while men in cars texted/read to a greater extent.

**Conclusion:**

The overall prevalence of cell phone use observed among motor vehicle drivers in Maputo city is in the upper range of those observed in high-income settings. Notwithstanding sex-related differences and variations by type of site and vehicle and coupled with the poor road safety conditions of the city – and of the country as a whole – and the imminent rise in cell phone ownership and internet connectivity, cell phone use at the wheel is a significant cause for concern and concerted actions are called for.

## Highlights

• The observed prevalence of 9.3 % is higher or in line with previous studies.
• Variations across sites were observed with the highest use (12.9 %) on straight roads.
• Differences were considerable across categories of vehicles.
• There were sex-related differences in the type of cell phone use.

## 1 Introduction

Cell phone use while driving has a history of about thirty years. The cell phone has become both a primary source of communication and an essential device for most people. Due to design improvements that increase personal convenience and have provided more available functions, and thanks to greater broadband internet access, individual behaviors have emerged that are difficult to refrain from at the wheel of a vehicle where they can be very harmful [1]. When asked, many of today's motorists acknowledge having an increasing dependence on their mobile phones [2]. There is a vast body of knowledge on the harmful consequences of cell phone use at the wheel and subsequent driver distraction. Studies have considered both hand-held and hands-free cell phone use [1] and have evolved as cell phone functions have diversified (see for instance [[3], [4], [5]]). Altogether, the accumulated evidence demonstrates that the risk of crash increases the greater the driver's visual, manual, and cognitive and acoustic distractions [5].

The assessment of cell phone use while driving is most typically based on self-reports or roadside observational studies. Reviews reveal that the prevalence indicated by survey data tends to be higher than that obtained through observational studies [1,6,7]. For instance, ten years ago, a review indicated that the percentage of drivers aged 18–64 years reporting talking on a cell phone while driving at least once in the past 30 days ranged from 21 % in the UK to 69 % in the United States, and that among those reporting having read or sent text or e-mail messages ranged from 15 % in Spain to 31 % in Portugal and the United States [7]. The figures have definitely increased since then to the extent that, recently, a prevalence of cellphone use of up to 93.4 % was observed among university students from Saudi Arabia, half of the users reporting using it often or always [8]. For their part, earlier reviews of observational studies, mainly based on data from rich nations, report a global prevalence of cell phone use while driving of around 1–7%–11 %, with similar levels in European countries specifically [1,6].

Both surveys and observational studies point to age- and sex-related differences. Younger drivers tend to use their phone more than their older peers [1,2,6,[9], [10], [11], [12]]. And men and women differ in the frequency, type, and duration of use [1].

Currently, many countries around the world prohibit the use of cell phone while driving, including 29 European countries and other large countries like the United States, Canada, Australia, and New Zealand. Cell phone use is also prohibited in several LMICs, including several on the African continent, e.g., Ghana, South Africa and Mozambique, the latter being the context of the current study. The laws are enforced unevenly harshly across countries [13] and monitoring cell phone use informs about levels of compliance across population groups and areas within a nation.

Monitoring cell phone use in LMICs is warranted not least in SSA where several significant communication changes are under way. Besides indications that the use is already a fact, despite variations across countries (see below), the rapidly rising level of cell phone ownership, reaching for instance 62 % in Mozambique [14], and advancements in the number of functions cell phones come with, the type of cell phone used is indeed set to change dramatically, from being predominantly feature phones to smartphones [14,15], as cross sectoral coalitions seek innovative solutions to boost the affordability of handset internet-enabled devices [16]. More and modernized phones will broaden the range of functions available and thereby increase the risk of inattention at the wheel. This will take place in a population of predominantly young and technologically savvy drivers [17]. Other reasons of importance pertain to the many features that make working with road safety more challenging in LMICs in general and in SSA in particular [18], notably the road traffic environment itself [19,20], the poor quality of the car fleet [21], and driver behavior [20].

In SSA, to the best of our knowledge, the first published empirical study on cellphone use is from Botswana in 2013 [22]. Since then, reports from several other countries have been published, assessing most typically different forms of distracted driving, cellphone use being then investigated alongside e.g., talking to other occupants in the car and eating/drinking/smoking. Invariably, cell phone use stands out as the most common form of distracted driving, in particular phone talking, with variations over time, and between and within countries. For instance, in the Botswanan study mentioned above [22], phone talking was reported among 31.2 % of the drivers observed in Gabarone and phone reading, in 8.2 %. In Cameroon [23], in a large city-wide study in Yaoundé, an overall use of handheld mobile device as low as 4.9 % was observed, varying not only by sex and age as in Botswana, but also by type of vehicle, site of observation and time of day.

Questionnaire-based studies, varying in scope, all point to preoccupying prevalence of cell phone use although with some variations: relatively lower levels reported in Zambia (37 % for self-reported use; [24]) and in Ghana (only 38 % of the commercial drivers reporting being compliant with the law [25]; slightly higher ones in Ethiopia ([26]; 69 % of commercial drivers disclosing having used their cell phone while driving over the past week); and very elevated ones in two Nigerian studies, with at about ten years of interval, respectively 87 % [27] and 97.8 % [28] of the drivers surveyed reported using their cell phone while driving in spite of they being aware of the law. It is only in the study from Cameroon [23] that the prevalence of cell phone use has been observed in varying circumstances (e.g., time of day and city area), and for different types of vehicles. Several differences were noticed, beyond expected sex- and age-related ones, and it is very likely that they could also be found in other countries from the region with higher levels of (self-reported) use alongside other risk behaviours [29].

This study, set in the context of Mozambique, a low-income country where a law prohibiting cell phone use while driving exists, was embarked upon to investigate the prevalence of cell phone use while driving among urban motor-vehicle drivers in the city of Maputo, and whether it varied by observation site, type of vehicle, and sex of the driver. Over half of the country's motorized vehicles (about one million in 2018 [30]), are driven in Maputo city.

## 2 Method

### 2.1 Study design

**Fig. 1:**
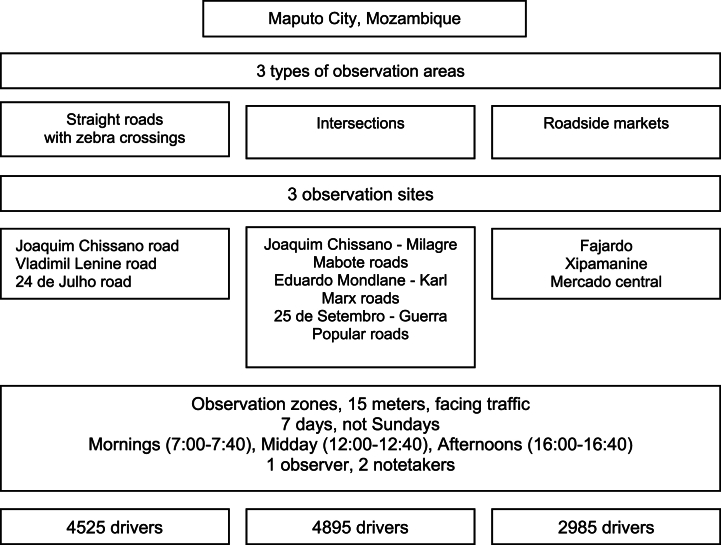
Schematic outline of the study design.

A non-invasive observational study was conducted for one month from 6th September to October 7, 2021. For a schematic outline of the design, please see Fig. 1.

### 2.2 Setting

The observations were conducted in Maputo city, the capital and largest city of the country. Maputo city had a population of 1 088 449 inhabitants in 2023, distributed over an area of 347.7 km^2^. Together with the surrounding municipalities of Matola, Boane and Marracuene, it forms the metropolis of the country. While Maputo city is largely urbanized, the residential and industrial growth experienced in recent years has taken place above all in Matola and Marracuene. As a result, the population density of the city remains low (an estimated 37 people/ha) [31]. Nonetheless, with the concentration of services in the center of Maputo, there is a heavy flow of people and vehicles commuting to and from the city daily, from Monday to Saturday, early morning to late afternoon [31].

**Fig. 2:**
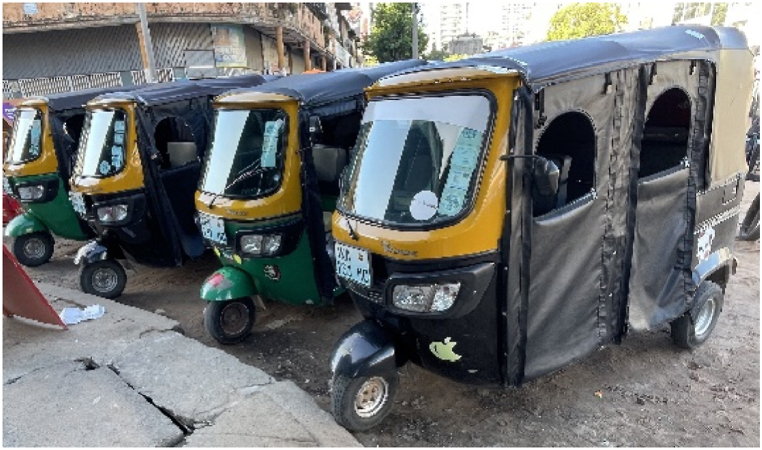
Three-wheeled *txopela*.

The rapid urbanization of the city coincided with a considerable increase in the number of vehicles, above all private cars [31] that are often in poor condition and lacking safety features (e.g., seat belts, working brakes, brake lights, windshield wipers). Additional vehicles to be found in the city include public buses and privately-owned minibuses used for public transportation, *txopela* (motorized three-wheelers used as a taxi; see Fig. 2) and trucks. Due to the slow development of the road infrastructure and services in the city that did not match the pace of motorization, the city is plagued by traffic congestion and its subsequently negative effect on public transport, economic growth, and environmental quality [31]. The streets mainly run at right angles, which means that intersections in Maputo city are numerous. Insufficient off-street parking also hampers traffic flow, not least in the city center. Cars are parked either on the road or on the sidewalk thereby reducing the road capacity [31]. Additional problems are caused by the maximum speed limit in Maputo (60 km/h), poor road conditions in general and a shortage of facilities e.g., sidewalks, crossings, and streetlights [31].

### 2.3 Selection of observation sites

**Fig. 3:**
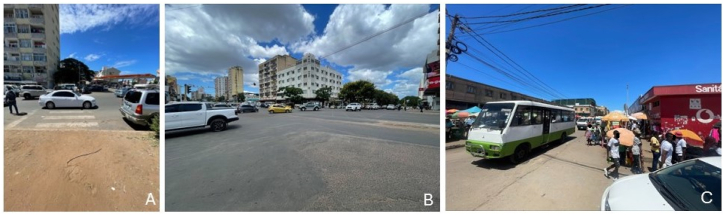
The three types of observations sites providing typical driving environments: A) on a straight road with zebra crossing, B) at an intersection, and C) by a roadside market.

For the observations, three types of sites were selected that reflect typical driving conditions requiring sharper attention on the part of the driver, including e.g. a mix of road users, rapid traffic flow, proximity to vulnerable road users and visibility issues. These sites were located on straight roads, at road intersections, and by roadside markets. The latter are common in Maputo and they are generally busy locations where motor vehicle drivers must be vigilant not to hit unprotected road users. Each of these three locations is illustrated in images A) - C) (Fig. 3).

Three specific observation areas were chosen for each type of site as follows.

• *straight road with zebra crossing* (all three roads must be used to enter or exit the city): Joaquim Chissano road; Vladmil Lenine road; and 24 de Julho road;
• *road intersections*: Joaquim Chissano and Milagre Mabote roads; Eduardo Mondlane and Karl Marx roads; and 25 de Setembro and Guerra Popular roads
• *roadside markets:* Fajardo, Xipamanine, and Mercado central.

It is of note that people drive on the left in Mozambique.

### 2.4 Data collection procedure

An electronic, online observation grid was used on cell phones or tablets, with date and time being automatically recorded and site of observation entered. The grid included 9 items that described among others whether and how a cell phone was used (coded as texting/reading, speaking/listening, unclear use, no use), the sex of the driver (male, female, unclear), and the type of vehicle (car, multi-person public transport vehicle (minibus and bus), *txopela* or truck.

At each of the nine selected sites, data were collected from a location that provided a relatively good view of the traffic flow. An “observation zone” of 15 m was determined, from the observation starting point (when a vehicle entered the zone) to the observer standing point (facing the traffic), a distance considered optimal for accurate observations. There were three data collectors, one observer and two notetakers. The data collectors were trained on several occasions before starting the observations. When it came to determining whether a cell phone was used and for what purpose, the following was agreed upon: 1) speaking/listening was coded when a driver could be seen holding a cell phone close to his ear or mouth; 2) reading/texting was coded in instances where the driver was writing on his phone or gave the impression of consulting information or reading. This implied that speaking/listening could include listening to music rather than having a conversation, but it was assumed that it entailed a similar kind of driving distraction. Likewise, reading could imply the use of a variety of functions/applications on the phone, from social media to GPS, but all were included in one. Finally, a person holding a phone in their hand was considered to be using the phone.

The observation periods lasted for 40 min each. Each period began with the random selection of a vehicle, followed by systematic observation of every fifth vehicle entering the observation zone. When a given data entry could not be determined by the observer, typically due to dark windows or fast-moving vehicles, it was coded as ‘not observable’. The period ended when the driver passed the observation standing point. Similar approaches have been employed in other studies (see for example 9 and 32).

At all sites, the observations took place on weekdays in the following time sequences: 07:00 to 07:40 (morning); 12:00 to 12:40 (midday) and 16:00 to 16:40 (afternoon) to capture a range of the population. Each site was observed over seven days, starting on different days and always excluding Sundays, when there is very little traffic in Maputo.

### 2.5 Participants

A total of 12 411 vehicles were observed (Table 1), of which 441 lacked information about the sex of the driver. The latter are included in the total column in Table 1 but excluded in later tables.

**Table 1:** Distribution of observed drivers by observation characteristics, stratified by sex (n = 12 411).

Observation characteristics	Men n = 9390		Women n = 2580		Total^a^ n = 12 411	
	N	%	n	%	n	%
**Observation Time**						
Morning	3345	35.6	1041	40.4	4521	36.4
Midday	2974	31.7	691	26.8	3849	31.0
Afternoon	3071	32.7	848	32.9	4041	32.6
**Observation Site**						
Straight Road	3545	37.8	869	33.7	4525	36.5
Road intersection	3538	37.7	1127	43.7	4895	39.4
Roadside market	2303	23.5	583	24.5	2985	24.1
Missing	4	0.04	1	0.04	6	0.1
**Vehicle Type**						
Car	6769	72.1	2532	98.1	9733	78.4
Truck	539	5.7	12	0.5	554	4.5
Minibus/bus	1716	18.3	24	0.9	1743	14.0
Txopela	334	3.6	0	0.0	336	2.7
Missing	32	0.3	12	0.34	45	0.4

a Including those 441 with unavailable information about sex.

### 2.6 Statistical analysis

We calculated the prevalence of cell phone use among the observed drivers (presented as percentage) with 95 % confidence intervals all drivers aggregated and stratified by sex. Sex-specific data were further disaggregated by site of observation and type of vehicle. A prevalence was not calculated for a sub-group of too small size (e.g., for women driving trucks or public transport minibuses/buses). Observations reported as “unclear” were excluded from the analyses. All statistical analyses were conducted in Stata version 16.

## 3 Results

### 3.1 Prevalence of cell phone use

Table 2 presents the prevalence of cell phone use overall and for men and women respectively, considering the type of vehicle (only cars for women) by observation site. The overall prevalence of cell phone use was 9.3 % (10.5 % among women and 9.0 % among men). It varied considerably across categories of vehicles, from 3.3 % for drivers of *txopela* to 10.3 % for car drivers. The overall prevalence varied also by site of observation, slightly over 7.1 % at road intersections and 7.5 % by roadside markets and as high as 12.9 % on straight roads, with minimal sex differences all vehicles aggregated, except for cars on straight roads (15.8 % and 12.1 % for women and men respectively).

**Table 2:** Sex-specific prevalence of cell phone use by type of vehicle, all observation sites aggregated and by site, expressed in percentages (%) with 95 % confidence intervals (95 % CI).

Type of vehicle	Road intersection n = 4587			Straight road n = 4248			Roadside market n = 2845			Total^b^ n = 11 680		
	n users^a^	%	95 % CI	n users^a^	%	95 % CI	n users^a^	%	95 % CI	n users^a^	%	95 % CI
**Men and women aggregated**												
**All vehicles**	**326**	**7.1**	**6.4–7.9**	**547**	**12.9**	**11.9–13.9**	**212**	**7.5**	**6.5–8.5**	**1085**	**9.3**	**8.8–9.8**
Car	291	7.6	6.8–8.4	463	14.8	13.6–16.1	182	8.7	7.5–9.9	936	10.3	9.7–11.0
Truck	11	5.5	3.1–9.7	14	6.7	4.0–10.9	7	5.2	2.5–10.5	32	5.9	4.2–8.2
Minibus/bus	20	4.9	3.2–7.5	65	8.1	6.4–10.2	21	4.1	2.7–6.1	106	6.1	5.1–7.4
Txopela	4	3.0	1.1–7.6	5	4.8	2.0–11.0	2	2.2	0.6–8.4	11	3.3	1.9–5.9
**Men**												
**All vehicles**	237	6.8	6.0–7.7	413	12.1	11.1–13.3	169	7.5	6.4–8.6	819	9.0	8.4–9.6
Car	203	7.4	6.5–8.4	333	14.4	13.1–15.9	139	9.1	7.7–10.6	675	10.3	9.6–11.0
Truck	10	5.1	2.8–9.2	14	6.9	4.1–11.3	7	5.3	2.5–10.6	31	5.8	4.1–8.2
Minibus/bus	20	5.0	3.2–7.6	61	7.7	6.1–9.8	21	4.1	2.7–6.2	102	6.0	5.0–7.2
Txopela	4	3.0	1.1–7.6	5	4.8	2.0–11.0	2	2.2	0.6–8.4	11	3.3	1.9–5.9
**Women**												
**All vehicles**	89	8.0	6.5–9.7	134	15.9	13.6–18.5	43	7.4	5.5–9.8	266	10.5	9.4–11.7
Car	88	8.0	6.5–9.7	130	15.8	13.4–18.4	43	7.5	5.6–10.0	261	10.4	9.3–11.7

a Number of cell phone users.

b Excluding the 441 drivers with missing information on sex and the additional 320 observations with missing information on cell phone use, vehicle type, observation site.

### 3.2 Type of cell phone use

Table 3 presents the sex-specific observed type of cell phone use all observation sites aggregated and stratified by type of site and vehicle. Overall, the differences between the prevalence of speaking/listening versus that of texting/reading were greater among women (52.6 % vs 36.1 %) than men (46.4 % vs 40.5 %) but men texted/read somewhat more than women (40.5 % vs 36.1 %) and women spoke more (52.6 % vs 46.4 %). On straight roads, the differences between men and women were small for both speaking/listening (46.5 vs 46.3 % for men and women respectively) and texting/reading (39.5 % vs 37.3 %). By roadside markets, the prevalence of speaking/listening among women exceeded that of men (58.1 % and 51.5 % respectively) just as it did but to a greater extent at road intersections (59.6 % vs 42.6 %) where men texted/read to a greater extent (44.7 % vs 32.6 % respectively).

**Table 3:** Sex-specific prevalence of type of cell phone use by mode of transport, all observation sites aggregated and by site, expressed in percentages with 95 % confidence intervals (95 % CI) (total number of observations = 1085).

Type of vehicle by sex^a^	Speaking/listening		Texting/reading		Undetermined	
	%	95 % CI	%	95 % CI	%	95 % CI
**All observation sites aggregated**						
**Men**	n = 380		n = 332		n = 107	
**All vehicles**	46.4	43.0–49.8	40.5	37.2–43.9	13.1	10.9–15.6
Car	45.2	41.5–49.0	41.0	37.4–44.8	13.8	11.4–16.6
Truck	61.3	43.4–76.5	32.3	18.3–50.3	6.5	1.6–22.4
Minibus/bus	52.0	42.3–61.5	37.3	28.4–47.0	10.8	6.1–18.4
**Women**	n = 140		n = 96		n = 30	
**All vehicles**	52.6	46.6–58.6	36.1	30.5–42.0	11.3	8.0–15.7
Car	53.3	47.2–59.2	35.2	29.7–41.2	11.5	8.2–16.0
**Straight road**						
**Men**	n = 192		n = 163		n = 58	
**All vehicles**	46.5	41.7–51.3	39.5	34.9–44.3	14.0	11.0–17.7
Car	45.6	40.4–51.0	39.6	34.5–45.0	14.7	11.3–18.9
Truck	64.3	37.6–84.3	28.6	11.1–56.1	7.1	1.0–37.1
Minibus/bus	49.2	36.9–61.5	37.7	26.5–50.4	13.1	6.7–24.1
**Women**	n = 62		n = 50		n = 22	
**All vehicles**	46.3	38.0–54.8	37.3	29.5–45.8	16.4	11.1–23.7
Car	46.9	38.5–55.5	36.2	28.3–44.8	16.9	11.4–24.4
**Road intersection**						
**Men**	n = 101		n = 106		n = 30	
**All vehicles**	42.6	36.4–49.0	44.7	38.5–51.1	12.7	9.0–17.5
Car	41.9	35.3–48.8	45.3	38.6–52.2	12.8	8.9–18.2
Truck	50.0	22.4–77.6	40.0	15.8–70.4	10.0	1.4–46.9
Minibus/bus	45.0	25.3–66.5	40.0	21.4–62.1	15.0	4.9–37.7
**Women**	n = 53		n = 29		n = 7	
**All vehicles**	59.6	49.0–69.2	32.6	23.6–43.0		
Car	60.2	49.7–69.9	31.8	22.9–42.3		
**Market by the road**						
**Men**	n = 87		n = 63		n = 19	
**All vehicles**	51.5	43.9–59.0	37.3	30.3–44.9	11.2	7.3–17.0
Car	48.9	40.7–57.2	38.1	30.4–46.5	12.9	8.3–19.7
Truck	71.4	32.5–92.9	28.6	7.1–67.5	0	–
Minibus/bus	66.7	44.5–83.3	33.3	16.7–55.5	0	–
**Women**	n = 25		n = 17		n = 1	
**All vehicles**	58.1	43.0–71.9	39.5	26.1–54.7		
Car	58.1	43.0–71.9	39.5	26.1–54.7		

a *Txopela* are not included due to small numbers.

Turning to the type of vehicles, in all three types of sites, men in trucks spoke/listened more than men in other vehicles while men in cars texted/read to a greater extent.

## 4 Discussion

This is the first relatively large roadside observation study on cell phone use while driving in Mozambique. The overall prevalence of cell phone use while driving observed among motor vehicle drivers in Maputo city was 9.3 %. This figure varied by observation site, being lower at road intersections and by roadside markets and higher on straight roads (from 7.1 % to 12.9 %). Although measurable only among men, there were also differences by type of vehicle, three times as high among car drivers (10.3 %) than among drivers of *txopela* (3.3 %). There was a male-to-female ratio of 1.17 in the overall prevalence of cell phone use while driving but speaking/listening was more prevalent than texting/reading for both sexes (52.6 % vs 36.1 % for women and 46.4 % vs 40.5 % for men). While there were small differences in sex-related type of use on straight roads, women spoke/listened more than men both by roadside markets (58.1 % and 51.5 %) and at intersections (59.6 % vs 42.6 %) and men texted/read more than women at intersections (44.7 % vs 32.6 %). At all three types of sites, men in trucks spoke/listened more than men in other vehicles. By contrast, men in cars texted/read to a greater extent.

The overall 9.3 % prevalence of cell phone use while driving observed in Maputo city is in the upper range of those observed earlier in high-income settings and aligns well with those reported in some countries of the same region like Zambia (9.6 %) [24] or Botswana (but here only for reading (8.2 %) but not talking (31.2 %) [22]. The overall prevalence is also lower than those found in all self-report studies from the region, including Zambia [24], Ethiopia (26; 69 % of commercial drivers); and Nigeria (87 % [27] and 97.8 % [28].

Finding differences according to site of observation is in line with previous literature. For type of road specifically, in Southern India for instance Ref. [32], the proportion of handheld cell phone use while driving on non-busy roads was almost two-fold more common than on busy roads. In our study, none of the roads qualified as “non-busy”. Rather, all of them could be quite busy but they entailed different road safety circumstances and challenges. The observed greater prevalence of cell phone use while driving on straight roads suggests more cautiousness in more complex road environments. An alternative explanation includes the slow-moving traffic on these roads at certain times of the day, that can be an incentive and provide an opportunity to use the phone. Overrating one's ability to deal with hazards or what is often termed “compensatory belief” [33] could also come into play. It is of note that the study from Cameroon mentioned earlier also found some differences by type of roads, but not all vehicles aggregated [23]. Although the results are hard to compare because that study aggregated all types of distracted driving at once, it was observed that light goods vehicles (LGV) and heavy goods vehicles (HGV) remarkable more use distracted driving on principal roads and busses on tertiary roads. Cellphone use was also more prevalent for those three groups of vehicles although not the most frequent form of distracted driving among them.

Age-related differences were not investigated (see limitations below) but sex-related ones were. There were differences in prevalence between all categories of vehicles aggregated and for cars specifically, the only category of vehicle where comparisons were possible. Men used a cell phone while driving more than women did and there were also differences in type of use. Sex differences have been found in previous studies from other – but not all – settings. When they occur, differences of this kind can be attributed to a range of factors, most of which will fall into social and cultural norms, to which risk-taking behavior belongs [34].

Further, it is noteworthy that the use of a cell phone at the wheel among male drivers peaked for cars and was lower in trucks and public transport vehicles (see also [24] and [35]). Truck drivers may have a far greater driving exposure than drivers of cars and those of public transport vehicles (minibuses or buses) and they are an acknowledged high-risk group for road traffic crash [1,6], a combination of attributes that makes them a group of particular concern for distracted driving. There are many laws and regulations in place that ban the use of cell phones at the wheel. But to be effective, those need to be informed by an understanding of the determinants of cell phone use, studies for which conceptual frameworks and theories have been proposed [36,37].

It is worth emphasizing that motorcycles are not a common mode of transport in Maputo, as it was not in Cameroon [23] which contrasts with some other low-and-middle income settings of other regions where it has been more studied like India [32] or Vietnam [38]. For the three-wheelers of Maputo, the use of a cell phone was so low that prevalence could not be reported, as opposed to trucks that appeared in similar proportions in their share of the total number of vehicles. Given the high risk of road traffic injuries related to two- and three-wheelers, this result may be considered as encouraging [39], although it is likely to reflect material deprivation in this working group.

### 4.1 Strengths and limitations

The strengths of this study pertain to its large sample of observations and the variety of sites, all representing typical traffic environments that drivers in Maputo City are exposed to. This made it possible to perform stratified analyses across observations sites with good statistical power. As the data at hand are overwhelmingly dominated by cars (about 8 in 10 observations overall and nearly all in the case of women), followed by about one in ten public transport vehicles (mainly privately own minibuses), it is difficult to determine how well the data represent cell phone use in all types of vehicles. But they do mirror the traffic environments and sites studied.

The methodology employed for this roadside observation study aligns well with previous studies in the field [9,32]. Being observational, the study is not affected by social desirability or recall biases, something that must be taken into account as Mozambique has a law prohibiting cell phone use while driving. As acknowledged earlier, a drawback of observation studies is their specificity in time and place, which is why decisions about when and where to observed are crucial for quality results. These aspects were considered thoroughly when developing the data collection plan.

Observational studies, although considered more objective, are still potentially prone to misclassification. In our study, around 2.5 % of the observations lacked data on cell phone use and an additional 3.5 % on the sex of the driver. This was mainly due to visibility issues, e.g. darkened widows or speed of the vehicle. Besides misclassification, there is a lack of precision in what the cell phone is used for as it is maybe difficult to determine what exact function (or application) of a cell phone is used. Therefore, just as in our study, most observations studies use broad categories of use.

The same observation and assessment principles were implemented at all types of site and locations, which indicates that underestimations, when occurring, affected all locations to the same extent. To minimize the risk of observer bias, those enrolled in the project were thoroughly trained, and regular data checks took place during the data collection.

The observations were conducted at different times during the day, and we did find some differences (results not presented) but not substantial ones that would point to a period being more critical than others. All three time periods selected were meant to represent distinct traffic flow but, in all of them, the use of cell phones is banned by law.

Our study is silent regarding age differences in cell phone use. Had it been studied, something that is difficult in observations, we would most likely have found some differences. Other studies have found that young people are more prone to use their cell phone than their senior peers [1,2,6,[9], [10], [11], [12]]. Further, there were no observations during evenings, at night and on Sundays. Observing at night and in the evening is not easy. And there are reasons to believe that, due to the increased risk of theft, drivers are less prone to use their phone during those periods, where the visibility can be reduced even for them due to poor street lighting. For its part, the decision not to observe on Sundays was mainly due to that day not being very representative of a usual weekday, with less commuting and much lighter traffic. Due to the non-invasive approach used in our study, it is possible that some motor vehicle drivers hid the use of their cell phone. Whether or to what extent this was the case is difficult to determine. Precautions were taken not to disturb the drivers (observers were instructed to observe discreetly, blend in, and avoid prolonged observation of a selected vehicle).

As the car fleet of Maputo city is different to those of other cities in the country situated in the north where motorcycles are far more common, the generalizability of the results to the rest of the country is uncertain (see also implications below).

### 4.2 Implications

The overall prevalence of cell phone use among car drivers in Maputo city, transformed into how many drivers at any one point in time in the city are using a cell phone at the wheel may put at risk the safety of all road users. Not only can this level be regarded as conservative, but it is likely to be increased both by an expected rise in the number of cell phone owners and by developments in the device itself, with a switch towards more (affordable) smartphones with a wider range of functions [15,40,41], giving rise to more sources of distracted driving.

This transition could perhaps provide an opportunity to reduce the use of cell phones at the wheel, but if the law and regulations in place are poorly enforced, the problem is unlikely to go away. It can instead be aggravated by motor vehicle drivers over-rating their ability to handle the consequences of their distractions at the wheel and under-rating the risks of cell phone use while driving [25]. Deterring the behavior will require the conception and implementation of well-contextualized strategies. These should perhaps even be customized as age-related differences can be expected in the prevalence of and motivations for cell phone use [2,37,42] and the population of Mozambique is predominantly young: 52 % of the population being under 18 years [43]. Those strategies will need to pay attention to the accessibility and spread of better performing cell phones and to the fact that they can be used in different forms, including portable, mountable, wearable, and inbuilt devices [44]. In countries like Australia and Canada, new rules have been introduced regulating all those forms of utilization while driving a vehicle or riding a motorbike [45].

An additional challenge is the high-level of coexisting unsafe driving behaviors among drivers, e.g., alcohol consumption and not wearing a safety belt, not least in LMICs [20]. For sure, there will be a need for concerted actions to discourage not only distracted driving, but also impaired driving, and other unsafe practices among car drivers – and their passengers.

## 5 Conclusion

The overall prevalence of cell phone use at the wheel observed among motor vehicle drivers of Maputo city is in the upper range of those observed earlier in high-income settings and in line with those observed in recent years in LMICs in SSA. Notwithstanding sex-related differences and variations by type of site and vehicle and, coupled with the poor road safety conditions of the city – and of the country as a whole – and the imminent rise in cell phone ownership and internet connectivity, cell phone use at the wheel is a significant cause for concern and concerted actions are called for to tackle the issue and promote responsible cell phone use.

## CRediT authorship contribution statement

**Alfredo Júlio Maposse:** Writing – original draft, Validation, Methodology, Investigation, Conceptualization. **Lucie Laflamme:** Writing – review & editing, Visualization, Supervision, Methodology, Conceptualization. **Franziska Fischer:** Writing – review & editing, Formal analysis, Data curation. **Jette Möller:** Writing – review & editing, Supervision, Methodology, Conceptualization.

## Ethical statement

The study was approved by the Ethical Committee of the Faculty of Medicine of the Eduardo Mondlane University (registration number: CIBS FM & HCM/003/2021). Informed consent was not obtained from the ones being observed, in order to not interfere with the traffic situation. Images from public roads are presented in a way so no individuals can be identified.

## Data availability statement

The data used in this study has not been deposited into any publicly available repository. Data will be made available upon request.

## Declaration of competing interest

The authors declare that they have no known competing financial interests or personal relationships that could have influenced the work reported in this paper.
